# Therapeutic Potential of Intravenous Immunoglobulin in Acute Brain Injury

**DOI:** 10.3389/fimmu.2017.00875

**Published:** 2017-07-31

**Authors:** Vivien Thom, Thiruma V. Arumugam, Tim Magnus, Mathias Gelderblom

**Affiliations:** ^1^Department of Neurology, University Medical Center Hamburg-Eppendorf, Hamburg, Germany; ^2^Department of Physiology, Yong Loo Lin School of Medicine, National University of Singapore, Singapore, Singapore

**Keywords:** acute brain injury, ischemic stroke, Fcγ receptors, sterile inflammation, treatment, intravenous immunoglobulin

## Abstract

Acute ischemic and traumatic injury of the central nervous system (CNS) is known to induce a cascade of inflammatory events that lead to secondary tissue damage. In particular, the sterile inflammatory response in stroke has been intensively investigated in the last decade, and numerous experimental studies demonstrated the neuroprotective potential of a targeted modulation of the immune system. Among the investigated immunomodulatory agents, intravenous immunoglobulin (IVIg) stand out due to their beneficial therapeutic potential in experimental stroke as well as several other experimental models of acute brain injuries, which are characterized by a rapidly evolving sterile inflammatory response, e.g., trauma, subarachnoid hemorrhage. IVIg are therapeutic preparations of polyclonal immunoglobulin G, extracted from the plasma of thousands of donors. In clinical practice, IVIg are the treatment of choice for diverse autoimmune diseases and various mechanisms of action have been proposed. Only recently, several experimental studies implicated a therapeutic potential of IVIg even in models of acute CNS injury, and suggested that the immune system as well as neuronal cells can directly be targeted by IVIg. This review gives further insight into the role of secondary inflammation in acute brain injury with an emphasis on stroke and investigates the therapeutic potential of IVIg.

## Sterile Inflammation of the Central Nervous System (CNS)

Acute tissue damage is known to trigger a highly conserved cascade of inflammatory events. This inflammation is vital for the immediate response of the host to invasive pathogens in the settings of acute infection and it is characterized by a rapid recruitment of neutrophils to the side of injury. Similar to the inflammation in response to microorganisms, trauma, ischemia, or chemically induced tissue damage elicit a rapid inflammatory reaction. Due to the absence of microorganisms, this inflammatory response is termed “sterile inflammation” (1). Sterile inflammation shows several similarities with innate immune responses toward microorganisms. Both microbially induced inflammation and sterile inflammation are characterized by the initial generation of danger-associated patterns (DAMPs), production of inflammatory cytokines as well as chemokines, and subsequent recruitment of leukocytes. In the CNS, sterile inflammation is mainly associated with an exacerbation of the tissue damage, induced by the initial event. In the case of cerebral ischemia, inflammation of the peri-infarct area contributes to a subsequent growth of the infarct core in the first days and is thereby contributing to a secondary worsening of the neurological outcome (2). However, at later stages inflammation might also be important for the resolution of the tissue damage and long-term recovery, even though these processes are currently only poorly understood (3). In addition to inflammation associated with acute injuries, there is also convincing evidence for the importance of chronic inflammatory processes in degenerative diseases of the brain, including Parkinson’s and Alzheimers disease (4).

### Development and Consequences of Inflammation in Stroke

#### Pathophysiology of Cerebral Ischemia

Ischemic stroke is a devastating disease and represents the most common cause of long-term disability in adults as well as the third leading cause of death in the western world. Due to the improving management of risk factors, the incidence of stroke in the western world has decreased over the past decades. Nevertheless, the prevalence has risen based on a reduced mortality (5). This shift will probably become even more prominent in the future due to improved treatment, but particularly on the basis of an increase in life expectancy and a net aging population. Considering the annual direct and indirect costs emerging for the treatment of stroke patients an enormous economic burden is emerging. Typically, an occlusion of a major artery, leading to disruption of blood supply, causes an ischemic stroke and the only treatment option is the early restoration of blood flow. Available treatment strategies include drug-induced thrombolysis as well as endovascular treatment with thrombectomy, but the major limiting factor is the onset-to-treatment time.

Focal disruption of cerebral circulation (ischemia) as well as the subsequent reperfusion contributes to brain injury. Initially, the restriction of blood flow leads to a rapid decrease of oxygen and glucose. Since brain tissue and particularly neurons are almost exclusively dependent on these substrates, they cease to function within minutes. Subsequently, activation of numerous signaling cascades, oxidative stress, mitochondrial dysfunction, and peri-infarct depolarization are initiated among other cellular events and cause neuronal necrosis as wells as apoptosis (6). Cells in the ischemic core are irreversibly damaged and quickly undergo necrosis. The surrounding tissue, the so-called penumbra, is still viable, but dysfunctional and extremely vulnerable. After the initial restriction of blood supply, reperfusion and reoxygenation lead to an aggravation of tissue damage particularly in the penumbra area, through the induction of a severe inflammatory, albeit sterile response (7).

#### Postischemic Inflammation

**Figure 1:**
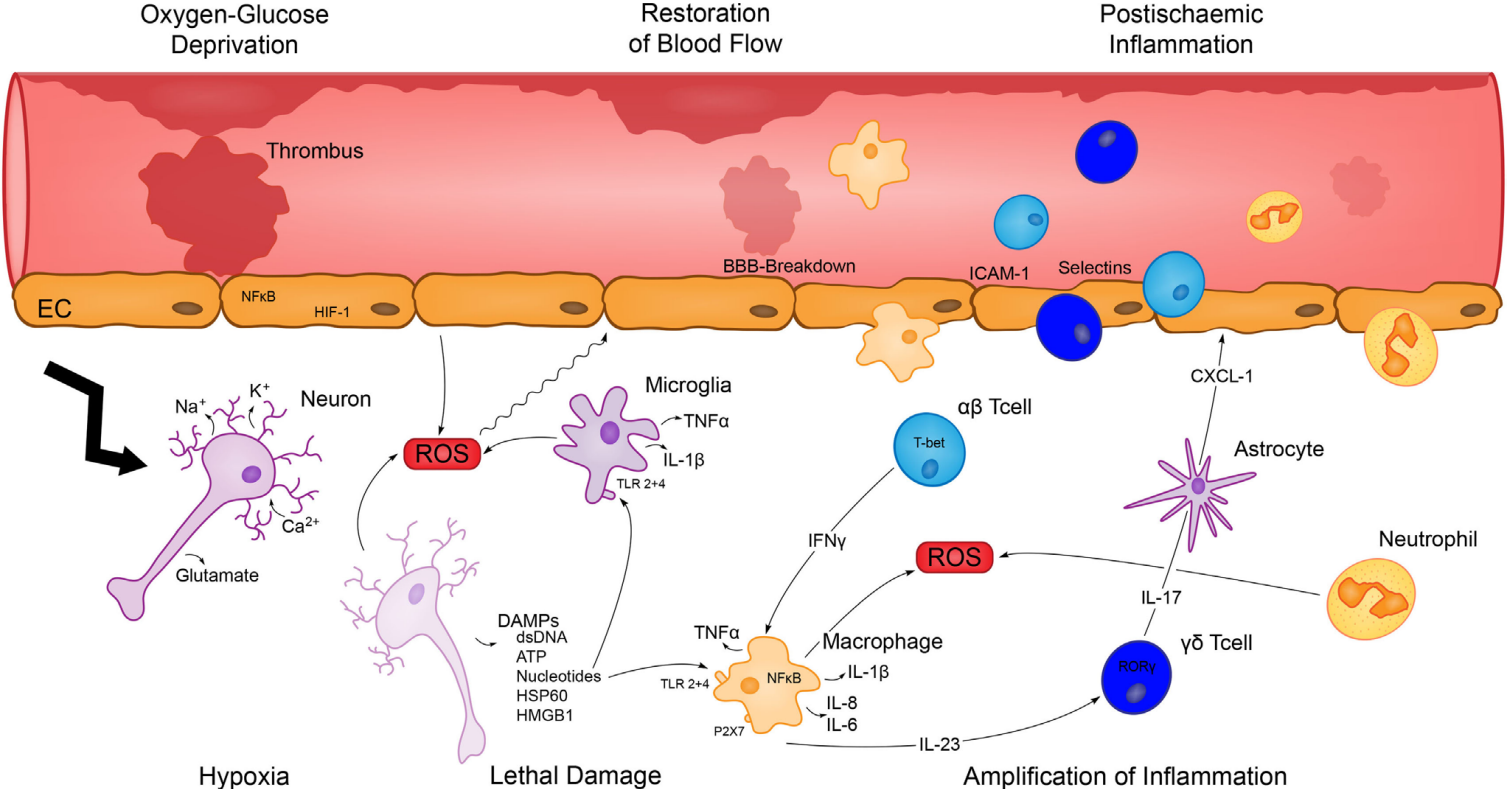
Depiction of the evolvement and amplification of postischemic inflammation. Hypoxia and glucose deprivation cause severe cell damage and dying cells release DAMPS and ROS, which activate resident immune cells. Subsequent production of inflammatory cytokines contributes to the breakdown of the blood–brain-barrier (BBB) and promotes the infiltration of cells of the adaptive as well as innate immunity, which cause a severe inflammatory response and deteriorate the initial brain damage.

Apart from early excitotoxic mechanisms promoting neuronal and glial cell death, the initial lesion enlarges within few hours and days after the ischemic event. This results in deterioration of the neurological deficit and poor functional outcome. A large number of reports support the hypothesis that inflammation is rather a cause than merely a consequence of brain injury. Infarct growth resulting from activation of the immune system by ischemia and subsequent reperfusion is recognized as a major element in all stages of the pathophysiology of ischemic stroke (as illustrated in Figure 1), including long-lasting regenerative processes (3). A reduction of infarct size as well as brain edema and improvement of neurological impairment could by achieved in the middle cerebral artery occlusion (MCAO) animal model by implementing various immunological alterations, such as using immuno-deficient mice, blocking antibodies against pro-inflammatory cytokines, and adhesion-molecules as well as anti-inflammatory treatment (8–13).

In response to the initial brain damage dying cells in the infarct core region release, DAMPs such as adenosin triphosphate (ATP) (14, 15) heat shock proteins (16), and high mobility group box 1 protein (HMGB1) (17), which activate pro-inflammatory membrane receptors, such as toll-like receptors (TLRs) and the receptor for advanced glycation end products (RAGE) in the penumbra region. Microglia are among the first immune cells to be activated by DAMPs after stroke (18). The rapid proliferation of resident microglia as well as subsequent infiltration of macrophages can be observed within the first hours following ischemia (19). Both cell types can produce inflammatory cytokines, such as tumor necrosis factor α (TNFα) and Interleukin (IL)-1β (8, 20) upon activation of TLRs, RAGE (21), and non-obese diabetic (NOD)-like receptor family pyrin domain containing protein (NLRP) 1 and NLPR3. It is well established that activation of TLR2 (22), TLR 4 (21), TLR 8 (23), and NLRP inflammasomes (24, 25) have been implicated in the context of postischemic inflammation. In addition, it was also shown that ATP as a DAMP activates the purinergic receptor such as P2X7 and contributes to postischemic infarct development (26, 27).

In addition to the hypoxia and ROS-induced breakdown of the blood–brain barrier (BBB), upregulation of endothelial adhesion molecules and pro-inflammatory cytokines, such as IL-1β and TNFα, promote further migration of leukocytes to the site of inflammation through the induction of chemoattractant signals (28). Lymphocytes only constitute a small fraction of infiltrating cells, but still play a prominent role in the evolvement of postischemic inflammation, although the temporal sequence does not correspond to established concepts of adaptive immunity. Mice deficient in lymphocytes have smaller infarcts (29) and specific depletion of the different T cell subpopulations T helper cells, cytotoxic T cells, and γδ T cells also revealed protective effects (12, 13). Whereas CD8 cells are important for perforin-mediated cytotoxicity (30), interferon γ (IFNγ) secreted by CD4 cells enhances the TNFα production of infiltrating macrophages (8). γδ T cells, in turn, produce large amounts of IL-17 in an IL-23-dependent manner (13), which synergistically with TNFα promotes neutrophil recruitment *via* the chemokine C-X-C motif ligand 1 (CXCL-1) (8). Conversely, administration of anti-IL-17 antibodies diminishes infarct size and improves neurological outcome. Just recently, the role of the inflammatory cytokine IL-21, which is mainly produced by CD4 cells, was also highlighted in the evolvement of postischemic inflammation (31). However, the role of regulatory T cells (Tregs) is more controversial. It was shown that the depletion of Tregs *via* the administration of anti-CD25 increased lesion size and neurological deficit (32), which led to the hypothesis that Tregs are protective in stroke and that their beneficial function depends on IL-10 (33). Contrary to these findings, Treg depletion through diphteria toxin injection in the DEREG mouse, a model to exclusively deplete Tregs, did not show an effect on lesion size (34). Furthermore, cells of the innate immune system are also involved in the processes of postischemic inflammation. The presence of DCs in the ischemic lesion, for instance, is a well-documented feature after stroke, although the functional relevance remains unknown so far (35). Neutrophils account for a substantial number of infiltrating cells (19) and blockade of the IL-17 axis diminishes neutrophil invasion and protects from ischemic stroke (8). Generally, preventing neutrophil migration to the brain has a beneficial effect (36) and neutrophils contribute to further brain damage by producing ROS, proteases, and inflammatory cytokines. Still, they also might have anti-inflammatory and neuroprotective functions and a more detailed understanding regarding their role in postischemic inflammation is needed (37). In contrast to the detrimental activation of the immune system in the CNS, a systemic immunosuppression caused by overactivation of the sympathetic nervous system is a common phenomenon following stroke (38). The clinical relevance is underlined by an increased frequency of pulmonary as well as urinary tract infections and can be partially attributed to a long-lasting lymphopenia and impaired cytokine production (39). Furthermore, a loss of innate-like B cells in the spleen, which can rapidly produce immunoglobulin G (IgG) and IgM in a T cell-independent manner and are important in the first-line of antibacterial defense, can be observed (40). Consistent with the loss of B cells murine and human studies have found that ischemic stroke can lead to decreased levels of IgG (41) and IgM (40).

Apart from the deleterious effects, the immunological processes are also a prerequisite for the structural and functional reorganization of the injured brain tissue (3). The inflammatory processes after stroke are self-limiting within the first week after the initial events. Microglia as well as infiltrating macrophages are important for the phagocytosis of dead cells and debris (18, 42). They are a source of tropic factors, growth factors (43), and IL-10 (44), thereby facilitating tissue repair. Furthermore, there is evidence that production of growth factors, such as insulin-like growth factor 1 (45) and vascular endothelial growth factor (VEGF) (46), are conducive to neuronal repair. Controversially, some molecules comprise destructive as well as protective capacity. Matrix metallopeptidase 9 (MMP-9), for example, not only exacerbates brain damage in the early phase after stroke (47) but also contributes to neurovascular remodeling and promotes poststroke recovery by converting pro-VEGF into an active form (48). Taken together, activation of the immune system contributes to poststroke inflammation and augments secondary brain damage after stroke. Furthermore, a systemic immunosuppression and an increased susceptibility to infections are observed after stroke. However, it is also important to note that postischemic inflammation may also be involved in regenerative processes. Therefore, it is important to dissect specific detrimental and protective mechanisms when developing new immunomodulatory treatment strategies.

#### The Postischemic Inflammatory Response in Human Stroke and Translational Approaches

Most of our current pathophysiological knowledge of the postischemic inflammatory mechanisms derives from murine experimental models employing the temporary MCAO mouse model. Despite the difficulty of obtaining postischemic human brain tissue, there is growing evidence of a similar critical inflammatory reaction following stroke in humans, which is based on histopathological postmortem studies and radiological findings. Repetitive magnetic resonance imaging (MRI) showed an enlargement of the ischemic lesion over time by 20% in selected patients (49) as well as the existence of a penumbra area (50). Migration patterns of infiltrating leukocytes were observed by single photon emission computed tomography and MRI. Neutrophils (51) as well as monocular phagocytes (52) infiltrated the ischemic hemisphere and also microglia were shown to be activated in the human brain following ischemic stroke (53). Brain autopsies validated these findings and showed an infiltration of neutrophils in the ischemic hemisphere within the first 48 h (54). Furthermore, infiltrating macrophages (52, 55), DCs, and T cells (56) could be found 3 days after stroke onset. Apart from the presence of immune cells, there is also evidence for poststroke inflammation. The pro-inflammatory transcription factor nuclear factor kappa-light-chain-enhancer of activated B cells (NFκb) and chemokines such as CXCL2 were upregulated (57). Furthermore, activated microglia could be detected in the penumbra area (58). Supporting the importance of IL-17, an enormous increase of IL-17 positive cells was found in the ischemic hemisphere (59), mostly in co-localization with infiltrated T cells (8). In addition, IL-17 messenger ribonucleic acid (mRNA) was found to be elevated in leukocytes from stroke patients (60).

In summary, there is substantial preclinical and clinical evidence for a pivotal role of postischemic inflammation in the pathophysiology of ischemic stroke and subsequent induction of further damage to the brain. Considering the limited time window of the available therapies, solely aiming at the restoration of blood supply, there is an urgent need of new treatment strategies. These should not only be applicable in a less restricted period of time but also target the inflammatory and regenerative processes after stroke. Despite the promising results in the mouse model, clinical trials testing neuroprotective and anti-inflammatory agents have largely failed so far (3, 61). The underlying cause of the translational roadblock can be attributed to the experimental model. First of all, different genetic backgrounds and significant difference in the composition and the function of the immune system exist between human and mouse (62). Furthermore, there are important varieties in brain morphology, anatomy of cerebral vasculature, and metabolism (6, 63, 64). Other relevant factors influencing the translation of preclinical studies concern the experimental model regarding the age of the animals and comorbidities, the stroke model in terms of distal versus proximal occlusion as well as transient versus permanent ischemia, outcome measurements, study quality, and selection of patients (65).

A recent example partly elucidating these issues is the investigation of the ability of natalizumab to reduce the detrimental effects of postischemic inflammation. Natalizumab is a monoclonal antibody against CD49d, an α4-integrin, preventing the migration of leukocytes into the brain in a very late antigen-4-dependent manner and is approved for the treatment of multiple sclerosis (MS). Anti-CD49d treatment was tested in different animals and distinct models of MCAO with varying periods of ischemia regarding the transient model. One group found a reduction of infarct size in the focal permanent model (30), in general resulting in small cortical infarcts, whereas another group could not reproduce these results (66). Comparable results were published for the transient model, where lesion size increases with the duration of ischemia. Short as well as extended periods of occlusion resulted in protection in some studies (30, 67, 68) but not exclusively (30, 66). In response to those deviating results a preclinical randomized controlled multicentre trial was initiated, which found that anti-CD49d treatment significantly reduced lesion size in the permanent model, but only when data from all centers were analyzed together, whereas there were no differences in the transient model (69). Nevertheless, a clinical study testing a single intravenous injection of natalizumab was conducted from December 2013 to April 2015, showing that natalizumab did not reduce infarct volume, but improved clinical outcome as measured by the modified Rankin Scale (mRS) (70).

Similar controversial is the published data on the immunomodulatory drug fingolimod, which acts as a functional analog of sphingosin-1-phosphate and, therefore, inhibits lymphocyte migration from the lymph nodes to the CNS. Conflicting data are published, mostly describing an impact of fingolimod (13, 71–74) but also challenging the effectiveness (75). Lately, however, two clinical pilot trials succeeded in showing a beneficial effect. Initially, they found in an open-label, evaluator-blinded fashion that fingolimod treatment is safe, attenuated the primary end point infarct growth and improved neurological outcome measured with the National Institutes of Health Stroke Scale and mRS in a cohort of 22 matched patients, who were not eligible for thrombolysis, given at a mean time of 22 h after symptom onset (76). The follow-up randomized, open-label, evaluator-blind multicenter trial investigated the effect of early fingolimod treatment in addition to thrombolysis (77). 47 patients, with 22 receiving fingolimod and rt-PA, were enrolled in the study and significant beneficial effects for the primary endpoints changes in lesion volume and extent of clinical improvement from baseline to day 1 as well as for the secondary endpoints extent of lesion volume growth and clinical improvement from day 1 to day 7 were observed. However, an unusual high rate of reperfusion of more than 60% was described in the second study and it needs to be considered that these studies have a proof of principle character and further multicenter, randomized, double-blind, and placebo-controlled trials will be necessary to confirm these results.

## FcγRs and Intravenous Immunoglobulin (IVIg)

Among many other immunomodulatory drugs, IVIg have been shown to be beneficial in experimental stroke in recent studies. IVIg contain polyclonal IgG and many different mechanisms of action have been proposed, of which the Fc fragment-dependent pathways seem to be of major significance. IVIg are established as a first-line therapy in different kinds of autoimmune disease. Although the mechanisms in stroke are not well understood so far, they possess promising therapeutic potential through neuroprotective and immunomodulatory pathways.

### Fcγ Receptors

**Figure 2:**
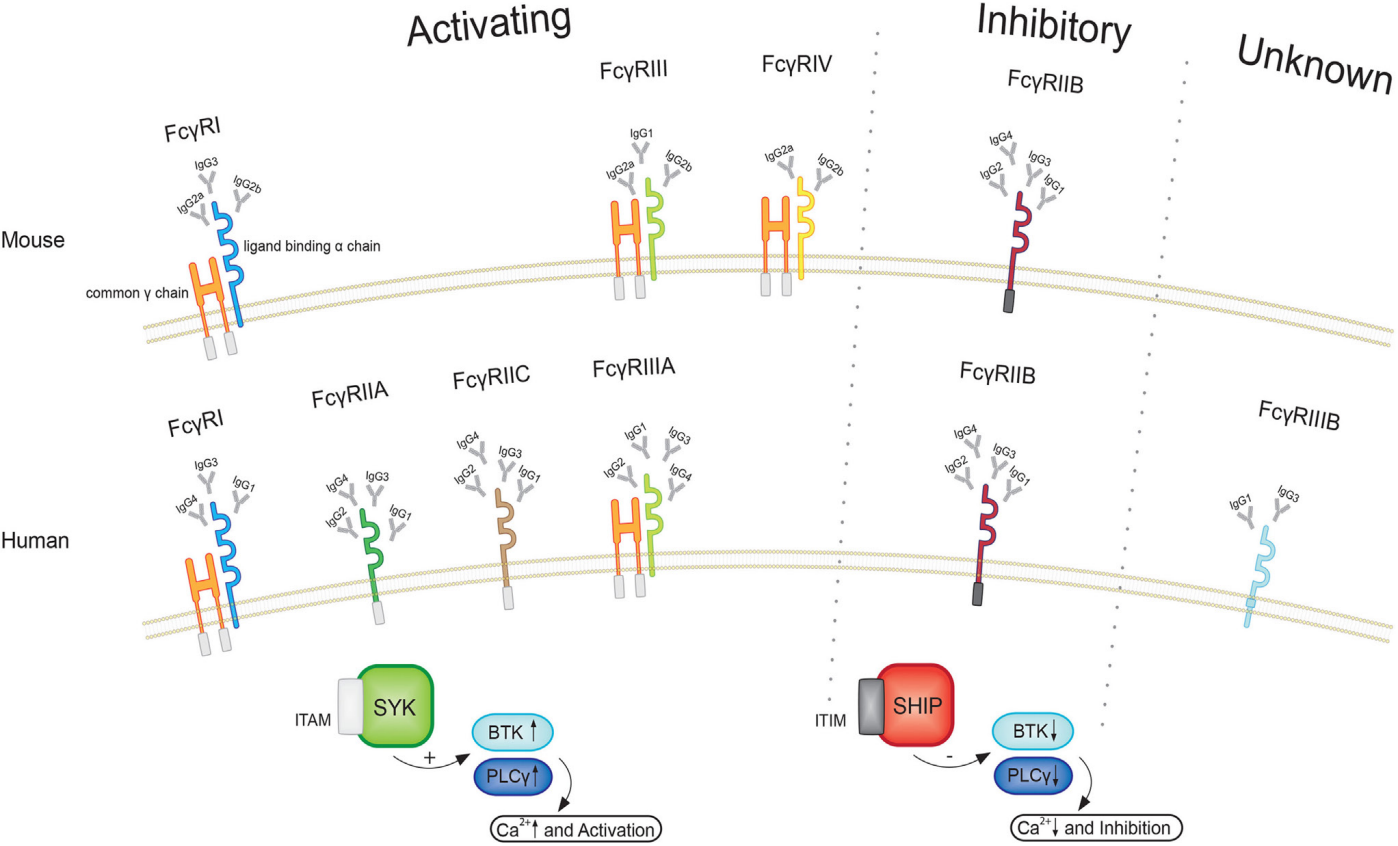
Illustration of the different FcγRs in human and mice. All activating FcγRs in mice as well as human FcγRI and FcγRIIIA express a common γ and a ligand binding a chain. After phosphorylation of the immunoreceptor tyrosin-based activating motif (ITAM), a signal cascade involving spleen tyrosin kinases (SYK), Bruton’s tyrosine kinase (BTK), and phospholipase Cγ (PLCγ) becomes initiated, leading to intracellular calcium influx and cell activation. Upon engagement of the inhibitory receptor, in turn, phosphorylation of the immunoreceptor tyrosin-based inhibitory motif (ITIM) leads to suppression of BTK and PLCγ. Since activating and inhibiting receptors are co-expressed and affect the same signaling pathways, the ratio of the different FcγRs sets a threshold for cell activation.

Fc receptors are found on the surface of a variety of cells and specifically bind to the Fc region of immunoglobulins (Igs). Four different subclasses of Fc receptors for IgG (FcγRs) have been identified in mice, including the activating receptors FcγRI, FcγRIII, and FcγRIV as well as the inhibitory receptor FcγRIIB (78). All FcγRs belong to the Ig superfamily. The activating receptors share a common γ-chain that comprises an immunoreceptor tyrosin-based activating motif (ITAM) (79) and express an individual ligand-binding α-chain, whereas the inhibitory FcγRIIB is a single chain receptor containing an immunoreceptor tyrosin-based inhibitory motif (as illustrated in Figure 2).

Besides microglial, endothelial, and mesangial cells as well as osteoclasts (78), FcγRs are particularly expressed by leukocytes. The majority of immune cells co-express activating and inhibitory FcγRs and the ratio of these receptors expressed by individual cells set a threshold for cell activation. Apart from distinct FcγRs, four different subclasses of IgG, in mice IgG1, IgG2a, IgG2b, and IgG3, with varying affinities toward the FcγRs are known. FcγRI shows high affinity and specificity for the different IgG isotypes, in contrast to FcγRIIB and FcγRIII that have lower binding capacities but recognize a broader spectrum (80). FcγRIV, in turn, seems to be the most important receptor for effector function of IgG2a and IgG2b (81). Moreover, it needs to be considered that the low-affinity receptors FcγRIIB, FcγRIII, and FcγRIV can only interact with multimeric IgG, which is present in immune complexes (ICs). This prevents unspecific binding, whereas FcγRI is saturated with monomeric serum IgG, but also requires ICs for activation (82). These kinetics suggest that the low-affinity receptors can regulate immunity more effective since the high-affinity binding to monomeric IgG of FcγRI hampers interaction with ICs (78). Apart from FcγRs IgG also binds to the neonatal Fc receptor (FcRn), which is expressed by vascular endothelium. FcRn prevents catabolism of IgG and is important for IgG half-life (83).

Triggered by the crosslinking of ICs with the α-chain of the activating FcγRs, the ITAM becomes phosphorylated, leading to the activation of members of the family of spleen tyrosin kinases (SYK) (78, 84). Subsequently, SYK-dependent phosphoinositides increase the activity of phospholipase Cγ (PLCγ) and Bruton’s tyrosine kinase (BTK) (78, 84) leading to an increase of intracellular calcium levels. Upon activation of FcγRIIB, in turn, the SH2-containing inositol polyphosphate 5-phosphatase (SHIP) hydrolyzes phosphoinositides (85). This events lead to a reduction of the activity of kinases, such as PLCγ and BTK, and, therefore, diminish the increase of intracellular calcium (78, 84). Hereby, activating as wells as inhibitory receptors affect the same signaling pathway and FcγRIIB exhibits important negative regulatory function in this manner. The loss or impairment of this inhibitory receptor can lead to autoimmunity as well as a prolonged immune response (86). The balance of activating and inhibitory receptors can be altered by the surrounding cytokine environment. The diverse role of Fc receptors in autoimmunity is elucidated by the fact that Fc receptor common γ-chain (FcRγ) knockout mice are resistant to the induction of autoimmune disease or show a milder course of disease, whereas FcγRIIB knockout mice often show worse outcome compared to wild-type mice (87–89). Mechanistically, it could be shown in nephrotoxic nephritis that DCs from FcγRIIB knockout animals show a more pronounced expression profile of cytokines associated with T cell activation that is absent in FcRγ-deficient mice (90). Furthermore, autoimmune-prone mouse-strains show a reduced expression of FcγRIIB, which is due to a promoter polymorphism in the Fcgr2 region (91). However, it needs to be considered that the FcRγ-chain is not only expressed by activating FcγRs but is also associated with a variety of other receptors, such as the T cell receptor (TCR)-CD3 (cluster of differentiation) complex, FcαR, FcεR, Nkp46, and IL3 (79, 92), when using the FcRγ knockout animals.

Human Fc receptors have a similar nomenclature and signaling pathways, but possess different expression patterns and binding affinities. So far, six different FcγRs have been identified. The high-affinity receptor FcγRI and the low-affinity receptors FcγRIIA, FcγRIIC, and FcγRIIIA are activating receptors, whereas FcγRIIB is inhibitory and the function of FcγRIIIB is yet unknown (93). The different IgG isotypes in humans are named IgG1, IgG2, IgG3, and IgG4, which bind to the different FcγRs in a concentration-dependent manner (94). Functionally, in humans IgG1 and IgG3 are the most pro-inflammatory IgG subclasses, whereas in mice IgG2a and IgG2b show a high inflammatory activity (78).

Taken together, humans and mice possess a similar repertoire of different FcγRs regarding their function and share common pathways. However, an important characteristic is the high rate of FcγR polymorphisms in humans (95), which affect binding affinities for IgG (94) and are associated with different kinds of autoimmune disease as well as immunological function (96).

### Immunomodulatory Effects of IVIg in Autoimmune Disease

Intravenous immunoglobulins are therapeutic preparations of polyclonal IgG, which are extracted from the plasma of thousands of donors. The different IgG subclasses are similarly distributed in IVIg preparations like in the blood of healthy individuals. Different mechanisms of action for IVIg in diseases models have been proposed. The Fc-mediated effects are most likely of major significance (97), which is supported by the fact that infusion of IgG preparations only containing the Fc fragment can protect mice as wells as humans from disease (98, 99). First of all IVIg can compete for activating FcγRs and, therefore, limit the access of activating ICs (100). In a similar manner, IVIg can inhibit complement deposition (101). The inhibitory receptor FcγRIIB seems to be essential for the protective effects, since protection by IVIg is lost in FcγRIIB-deficient mice and FcγRIIB is upregulated following IVIg treatment (88, 99). Mechanistically, FcγRIIB signaling can suppress B cell-mediated T cell-dependent immune responses (102). Specific intracellular adhesion molecule grabbing none-integrin receptor 1(SIGN-R1) and its human homolog dendritic cell-specific intracellular adhesion molecule-3 grabbing non-integrin, which is among others expressed by macrophages (103), have also been proposed to be important in IVIg function mediated by FcγRIIB. These two receptors can directly recognize specific sialic acid rich isoforms of IgG, which leads to an up to tenfold increase of effectiveness of IVIg (104) and induces a non-inflammatory phenotype in a specialized subset of macrophages. Conversely, macrophage colony-stimulating factor 1 (M-CSF-1)-deficient mice also loose IVIg protection, which might be due to a lack of M-CSF-1-dependent regulatory SIGN-R1 expressing macrophages (105). Furthermore, inhibition of BTK, a kinase downstream of FcγRs, whose activity is reduced upon FcγRIIB activation, leads to reduced mature IL-1β in the context of inflammation (25), as well as preventing IL-1β, IL-17, IFNγ, and TNFα production upon FcγR stimulation (106).

Another proposed functional pathway is the ability of IVIg to neutralize anti-ideotypic antibodies in a F(ab)^2^ fragment-dependent manner and, therefore, protect from disease (107). Other F(ab)^2^ fragment-dependent mechanisms include blockade of specific receptors, such as the Fas receptor *via* anti-Fas antibodies (108), the binding of cytokines such as IL-5 as wells as granulocyte macrophage colony-stimulating factor (109) and the inhibition of TCR-mediated T cell proliferation (110). In addition, IVIg do not only hemper T cell proliferation but can also influence the differentiation of T helper cells into Th17 cells *via* interference with the retinoic acid-related orphan receptor C (111) and can induce a shift toward a Th2 phenotype in childhood ITP and women with recurrent spontaneous abortion (112, 113). Furthermore, there is evidence that IVIgs increase the expression of IL-10 as wells as TGF-β in T regulatory cells (114) *via* specific epitopes in the Fc region of IgG (115). Interestingly, IVIg were also able to bind to peripheral blood T cells, which do not express FcγRs, through yet unknown receptors (116) and can reduce the production of inflammatory cytokines, such as IL-2, IL-3, IL-4, IL-5, IFNγ, and TNF in *in vitro* setting (117).

Taken together IVIg can act through a variety of different pathways. Apart from Fc and F(ab)^2^ fragment-dependent effects, IVIgs can either indirectly or even directly address cells that do not express FcγRs. The individual roles and mechanisms need to be explored individually in the different autoimmune diseases, also considering that there are probably joint effects.

### FcγRs and IVIg in Neurological Disease

#### Expression of FcγRs in the CNS

There is growing evidence for a pivotal role of Fc receptors in the pathophysiology of disorders of the CNS, but the existence of the distinct FcγRs within the cell types of the CNS are still not fully explored. Especially the functionality and expression profile of FcγRs in neurons remains controversial. Nevertheless, mRNA of all FcγRs has been found in primary mouse superior cervical ganglion cultures and an intracellular calcium increase upon stimulation with IgG could be detected (118). Considering that FcγR signaling in immune cells causes an intracellular calcium increase *via* SYK and subsequent activation of BTK and PLCγ, it is conceivable that the same pathway is enabled in neurons. FcγRI has also been found on dorsal root ganglions, similarly causing intracellular calcium increase (119), whereas FcγRIII was expressed by primary neuronal cell cultures (120). FcγRIV could be detected in the hippocampal area and temporal cortex in mice brain (121). Likewise, the inhibitory receptor FcγRIIB is expressed by neurons (122) and has an important function in the cerebellum during development (123). Little is known about the regulation of these receptors in neurons, but elevated mRNA levels of all FcγRs could be found in response to an increase of intracerebral IgG in a model of experimental hypercholesterolemia (121).

Microglia express all FcγRs (124) and their expression levels are upregulated in response to inflammation and cytokines, such as IFNγ, TNF, and IL-1β (125–128). Upon stimulation with monoclonal antibodies against the FcRγ-chains I, IIA, III, but not IIB human microglia can produce inflammatory cytokines such as macrophage inflammatory protein 1α (129). Furthermore, it could be shown that the expression of FcγRIV increases with age and upon lipopolysaccharide stimulation (130). An upregulation of all FcγRs in models of chronic neurodegeneration in response to inflammatory stimuli was also observed (131), whereas FcγRI and FcγRIIb were downregulated on microglia of AD patients after immunotherapy (132, 133). Apart from microglia, astrocytes and oligodendrocytes are also known to contribute to postischemic inflammation, but only limited data on the expression of FcRs are available in these cell types. Astrocytes have been reported to express FcγRI and FcγRIIB (134), whereas oligodendrocyte precursor cells (OPCs) express FcRγ as well as the alpha chain of FcγRI and FcγRIII (134). Stimulation with anti-FcRγ as well as IgG induces differentiation into myelinating oligodendrocytes, suggesting that FcγRs are expressed on cells of the oligodendrocytes lineage and are important for myelination. Conversely, the FcRγ mice show hypomyelination (135).

In summary, there is much evidence that all FcγRs are expressed in neurons, microglial as well as other glial cells of the CNS. Particularly the inhibitory receptor FcγRIIB could be found on all cell types. Their existence seems to be not exclusively important in immunological processes but also in the context of development of the CNS.

### Immunomodulatory Effects of IVIg in Neurological Disease

Intravenous immunoglobulins are established as a first-line therapy in a variety of neurological disease. They are used in the treatment of CIDP, Guillain–Barré syndrome (GBS), myasthenia gravis, and inflammatory myopathies as well as in autoimmune encephalitis and neuromyelitis optica (136, 137). CIDP, for example, is a heterogeneous autoimmune-mediated inflammatory demyelinating disease of peripheral nerves and several clinical trials showed that IVIg are beneficial (138). The pathophysiology of disease remains unknown; but in a subset of patients, it appears to be mediated by IgG-autoantibodies against myelin. IgG isolated from this group of patients was able to induce disease in rats (91), suggesting that IgG can play an important role in the development of CIDP. Furthermore, the NODmouse strain, which has a promoter polymorphism in the Fcgr2 region (139), can develop spontaneous autoimmune peripheral polyneuropathy under certain circumstances (140, 141). Conversely, FcγRIIB expression is impaired in B cells of patients with CIDP but is upregulated following IVIg treatment (142). Likewise, for GBS, another disease of the peripheral nervous system (PNS), IVIg represent an established therapeutic regime. In approximately 50% of patients with the GBS, autoantibodies against gangliosides can be found (143). One of the proposed mechanisms of IVIg action in GBS as wells as CIDP is the presence of anti-ideotypic antibodies that are able to bind and neutralize pathogenic autoantibodies (144). Another exemplary IgG-mediated neurological disorder is myasthenia gravis, in which autoantibodies against the acetylcholine receptor are produced in a T helper cell-dependent manner. Mechanistically, IVIg protection in myasthenia gravis is also most likely promoted by anti-ideotypic antibodies.

Apart from the efficacy to treat PNS affecting diseases, data for IVIg on diseases of the CNS is more controversial. Although beneficial effects and potential therapeutic pathways have been observed in the mouse model of MS (145) and AD, for example, translation into the human system remains difficult so far. A major limiting factor in the context of diseases of the CNS is the BBB, which controls access of IVIg to the brain. In the healthy brain, IgG is present in small amounts and is able to cross the BBB in a controlled manner through yet unknown mechanism. The clearance from the CNS is mediated by the FcRn (146), which is expressed by brain endothelial cells (147, 148). In the context of inflammation, when the BBB is disrupted, IVIg are able to enter the brain in a less restricted manner. Accordingly, following administration of IVIg in a model of experimental stroke, increased intracerebral IgG was observed in the ischemic brain (147, 148). IVIg is also able to cross the intact BBB in a saturation-dependent process and were found to co-localize with neurons as well as endothelial cells (149). Interestingly, administration of IVIg reduced the amount of endogenous IgG in this model, suggesting a competition for brain access. A positive impact of IVIg on the integrity of the BBB has also been reported, since IVIg treatment was able to prevent BBB-breakdown in sepsis (150).

For MS, which is suggested to be primarily a T cell-mediated disease, there is experimental evidence from the experimental autoimmune encephalitis (EAE) model that treatment with IVIg is beneficial. It could be shown that IVIg decreased the production of inflammatory cytokines such as IFNγ and TNF (144, 151) on one side and led to an expansion of peripheral T regulatory cells and subsequent suppression of conventional T helper cells in an Fc-fragment independent manner on the other side (152). Apart from the impact on the cells of the adaptive immunity, there is also evidence for a direct involvement of FcγRs. It could be shown that FcRγ-deficient mice develop milder EAE (89), although this effect was attributed to γδ T cells that use this chain in other receptors than the FcγR. In addition, the FcRγ can be detected on OPCs in remyelinating plaques in MS as well as on microglia in inactive plaques (153). Despite the beneficial action in the experimental setting, the effect of treatment in humans is controversial. Some small studies were able to show protection, whereas a large multicentre, randomized, double-blind, placebo-controlled trial failed to reproduce the previous results (154).

## IVIg in Acute Brain Injury

### Current Knowledge of Fc Receptors and IVIg in Stroke

Only little is known about the role of FcγRs in the context of postischemic inflammation, but various experimental studies emphasize a promising therapeutic effect of IVIgs in the *in vivo* stroke model as well as in *in vitro* settings by reducing infarct size and improving neurological outcome. The direct mechanisms remain unknown so far. First of all, it could be shown in the MCAO model that the common γ-chain knockout, which lacks all activating FcγRs, is protected from stroke (155). Compared to WT mice, the common γ-chain-deficient mice showed significant reduction of infarct volume at 24, 72 h, and 14 days after stroke as well as an improved neurological outcome after being subjected to 60 min of transient focal ischemia. Mechanistically, it was assumed that FcγRs are important for the activation of microglia and induction of the inducible nitric oxide synthase (iNOS). Hence, microglia of the common γ-chain KO mice expressed fewer ionized calcium-binding adapter molecule 1, a protein that is upregulated in microglia upon activation, as well as less iNOS in immunohistochemistry and on protein level.

In addition to the effects mediated by inhibition of the activating FcγRs, it was recently shown that IVIg treatment likewise protects the brain from ischemia-induced reperfusion injury and the subsequent inflammatory response. Administration of IVIg, 3 h post reperfusion significantly reduced the amount of infiltrating leukocytes 24 h after MCAO, which were identified as CD45 high cells in flow cytometry (156). In line with recent preclinical studies showing a beneficial effect of the α4-integrin-inhibitor natalizumab in experimental stroke, the reduction of infiltrating leukocytes by IVIg could be mediated in an α4-integrin-inhibitor-dependent manner as it has been shown in the EAE model (157). Of note, this study concluded that IVIg treatment is even detrimental in stroke, since they found more leukocytes occupying pial vessels, which is thought to be due to platelet-mediated pro-adhesive effects. Still, it needs to be considered that they did not examine the actual number of infiltrating cells.

Furthermore, IVIg treatment of primary neuronal cultures subjected to oxygen and glucose deprivation, an *in vitro* model of ischemic stroke, inhibited upregulation of TLR2, TLR4, and TLR8 (158). These findings could also be reproduced *in vivo* where IVIg administration after transient MCAO significantly reduced ischemia-induced upregulation of TLR2, TLR4, and TLR8. The authors also observed an IVIg-dependent suppression of HMGB1-mediated TLR activation, which is released by dying cells as a danger signal in the context of ischemic stroke. In addition, it was found that IVIg attenuated the ischemia-induced increase of complement factor C3b, which is known to contribute to ischemic injury (159) and among others upregulates the intracellular adhesion molecule 1 (ICAM-1) *in vivo* and *in vitro*. In line with these findings, IVIg also specifically affect endothelial cells and diminish the upregulation of VCAM-1 and ICAM-1 in *in vitro* settings (160).

Another immunomodulatory IVIg mechanism includes the suppression of the NLPR1 and NLPR3 inflammasome-mediated neuronal cell death (24, 25). Treatment of primary neuronal cultures with IVIg subjected to simulated ischemia as well as mice subjected to MCAO, reduced levels of inflammasome components such as NLRP1, NLRP3, and apoptosis-associated speck-like protein containing a caspase recruitment domain (ACS). This, in turn, led to a reduction of caspase-1 and mature IL-1β as well as IL-18. Moreover, it was shown that selective inhibition of BTK with ibrutinib, whose activity is reduced after engagement of the inhibitory receptor FcγRIIB, diminishes ischemic injury by decreasing inflammasome NLPR3 activity and, therefore, conversion of pro-IL-1β (25). Conversely, the inhibition of SYK, a kinase upstream of BTK was also able to decrease lesion size in the MCAO model (161). Interestingly, IVIg protection in other autoimmune disease is lost in M-CSF-1-deficient (op/op) mice. Apart from an enormous reduction of microglia proliferation, these M-CSF-1-deficient mice show an enhanced sensitivity to ischemia-induced neuronal injury and cell death (162). M-CSF-1 overexpression, in turn, leads to microglia proliferation, which does not show a difference in phenotype regarding the M1/M2 model, but shows altered immune responses (163). Importantly, M-CSF-1 treatment of mice subjected to MCAO decreases infarct size (162) and the presence of M-CSF-1-dependent macrophages correlates with an increased expression of FcγRIIB (164).

Apart from ameliorating the inflammatory response after stroke the protective capacity of IVIg includes the induction of neuroprotective pathways. For instance, neuronal structure was more intact and had less ischemia-associated alterations on a histopathological level in IVIg-treated rats (165). IVIg treatment of primary neuronal cultures subjected to simulated ischemia for 12 h significantly reduced protein levels of factors involved in neuronal cell death like the phospho-SAPK c-Jun NH^2^-terminal kinase (p-JNK) and phospho-p65 NFκB and inhibited the loss of the neuronal marker microtubulin associated protein 2 (MAP2) (147, 158). These findings could be confirmed by immunoblots and immunohistochemistry, respectively, *in vivo* in the transient MCAO model. Furthermore, IVIg could prevent simulated ischemia-induced endothelial disintegration in a brain endothelial cell line and increased the protective protein B-cell lymphoma 2 produced by these endothelial cells as well as by neurons (156). In line with these findings it could be shown that ischemia-induced decrease and reduced phosphorylation of low-density lipoprotein receptor-related protein 1, which is abundantly expressed by neurons, can be inhibited by IVIgs and, subsequently, prevents activation of cell death signaling proteins as NFκB and p-JNK (166).

**Figure 3:**
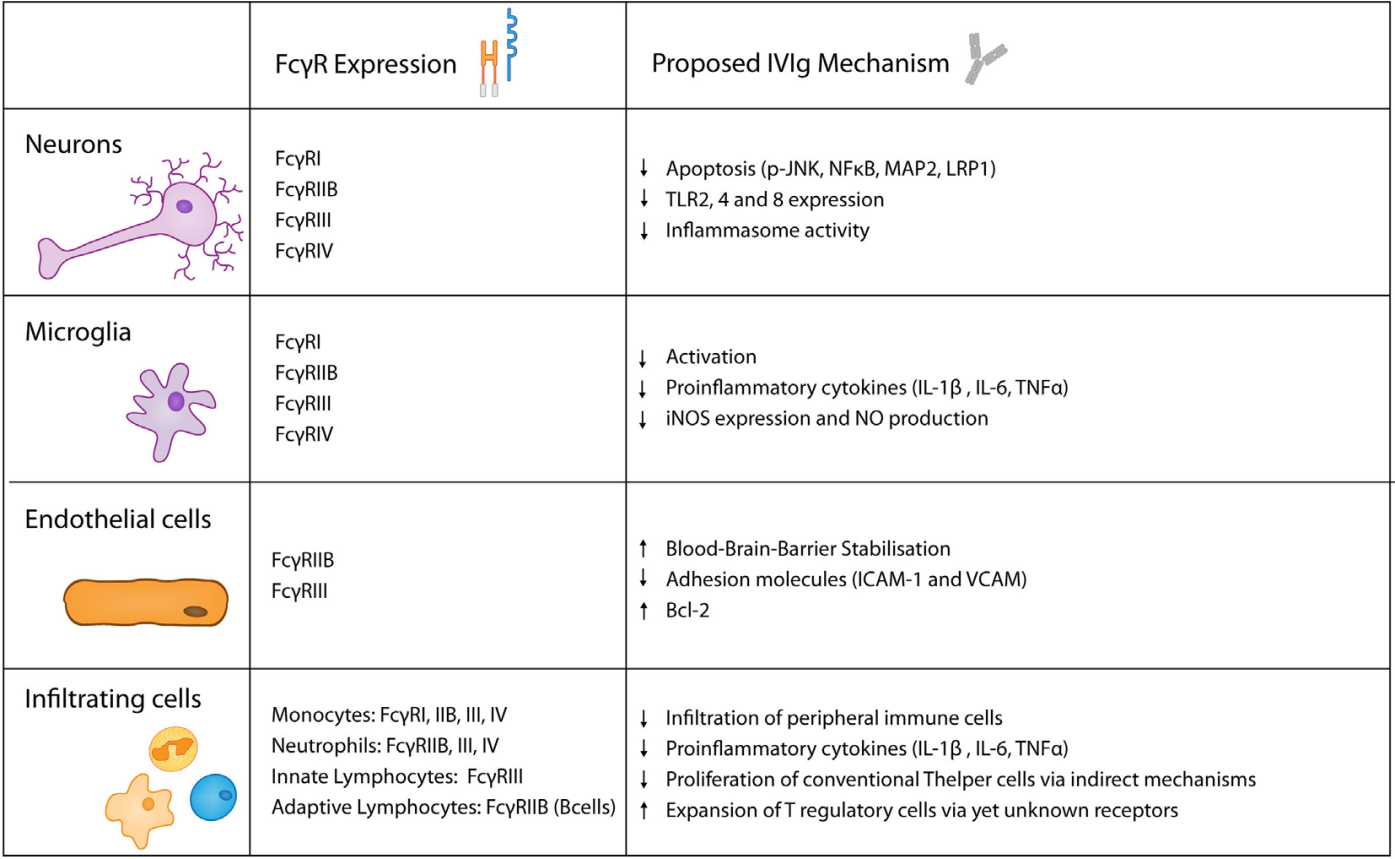
Keyplayers in acute brain injury and their expression of FcγRs as well as their proposed role in intravenous immunoglobulin (IVIg)-mediated protective effects.

Apart from the already described pathways, there are multiple other conceivable mechanism how IVIg facilitate protection in the context of ischemic stroke (Figure 3). They could inhibit complement deposition and induce a regulatory phenotype in macrophages in an Fc-fragment manner, as well as targeting the inhibitory receptor FcγRIIB, subsequently supressing T cell-mediated immune responses. Similary, the production of inflammatory cytokines, such as IL-1β and TNFα, could be diminished in FcγRs expressing cells. In addition, the F(ab)^2^ fragment could block receptors such as Fas (167) or inhibit TCR-mediated T cell proliferation. Also indirect effect such as increased IL-10 and TGF-β expression as well as a reduced production of inflammatory cytokine by T cells need to be considered. Ultimately, it remains unsolved wheter IVIg primarily operate *via* the Fc or F(ab)^2^ fragment, or if indirect mechanisms such as T cell inhibition predominate.

### IVIg in Other Models of Acute CNS- and Ischemia-Reperfusion Injury

Besides experimental evidence for positive IVIg effects in postischemic inflammation, there are other models of acute CNS injury in which IVIg have been shown to be beneficial. Similar to inflammatory processes following ischemic stroke, neuroinflammation exacerbates the tissue damage in acute spinal cord trauma (168). Accordingly, several hallmarks of sterile inflammation have been observed following trauma: (i) microglia activation; (ii) upregulation of proinflammatory cytokines, including IL-1β, TNFα, and IL-6 as well as ROS and MMP-9; and (iii) infiltration of neutrophils, monocytes, and lymphocytes. The importance of the local inflammation is underlined by studies showing that immunosuppressive treatment approaches with steroids have favorable effect on functional outcome. Nevertheless, the disadvantage of unspecific immunosuppressive agents is demonstrated by studies showing an increased risk of infection following steroid treatment in models of traumatic spinal cord injury. Consequently, more specific immunomodulatory treatment strategies are needed. The feasibility of immunmodulatory treatments in acute spinal cord injury was recently demonstrated by Gok et al. (169), describing that the treatment with IgG had significant beneficial effects on motor function in a rat model. Furthermore, electron microscopy revealed a significant decrease of intraneuronal vacuoles, as an indicator for more preserved neuronal ultrastructure. Another study on IgG in acute spinal cord injury also showed reduced scar formation and tissue preservation on a histopathological level (170). In addition, the authors detected a reduction in proinflammatory cytokines, such as TNFα, IL-1β, and IL-6 as well as MMP9, and showed that the IgG is able to enter the injured spinal cord while it mainly co-localized to astrocytes. Furthermore, protective IVIg effects were associated with reduced numbers of infiltrating neutrophils as wells as a diminished MPO activity. Overall, the reduction of inflammation showed improved functional recovery assessed by neurobehavioral test as well as significantly enhanced conduction velocity in electrophysiological measurements.

Sterile inflammation is also a well-known feature after traumatic brain injury (171). As a consequence of the initial trauma a tremendous release of DAMPs can be observed, which induced production of proinflammatory cytokines and infiltration of various immune cells. Incidentally, it was found that IgG significantly improved motor test scores compared to saline and reduced MPO activity, as it was used as an additional vehicle control in a study investigating the effect of ICAM-1 blockage in traumatic brain injury (172). Furthermore, it could be shown that IVIg treatment stabilized the BBB and reduced edema formation (173). These effects were accompanied by reduced amounts of IL-6 upon IVIg administration as well as less infiltrating macrophages and increased neuronal density. Moreover, endothelial protection by IVIg was detected in a model of subarachnoidal hemorrhage (174).

Intravenous immunoglobulin have also been investigated in other models of ischemia-reperfusion injury and a protective effect on the evolving inflammatory cascades has been observed. In mesenteric ischemia, a condition that can be associated with hypovolaemic shock, sepsis, and cardiac arrest (175), pretreatment with IVIg reduced complement-mediated tissue damage (176). IVIg also significantly reduced mucosal injury on a histopathological level and diminished C3 deposition in the intestinal mucosa. Interestingly, the number of infiltrating leukocytes was not altered by IVIg administration. Similarly, in a model of liver ischemia, sinusoidal congestion and cytoplasmatic vacuolation were diminished in IVIg-treated mice (177). These effects were associated with a reduced mortality in the IVIg-treated group. Taken together, there are multiple pieces of evidence from experimental studies that IVIg have significant protective effects on acute injuries of the CNS and other organs, in which sterile inflammation is part of the pathology.

### Implications for the Clinical Use of IVIg in Acute Brain Injury

Reflecting the available preclinical data on IVIg in acute brain injury, it seems promising to also use IVIg in a clinical setting. However, possible rheologic disadvantages need to be considered. It does not appear intuitively that an agent, which could possibly deteriorate perfusion due to the high viscosity, is suitable to treat diseases with reduced blood flow and impairment of microcirculation. Indeed, IVIg-related thrombosis has been described in the literature sporadically (178). Nevertheless, the positive impact of IVIg seems to predominate these negative effects. Overall, these drawbacks might be overcome in future studies, if specific therapeutic effects can be attributed to single IVIg fractions, thereby allowing to reduce the necessary dosage.

Considering that peripheral immunosuppression and an increased risk of urinary tract as well as upper airway infections is a common epiphenomenon after stroke IVIg treatment shows another promising potential. Apart from reducing the inflammatory reactions after stroke, IVIg administration could also compensate for the transient decrease of IgG after stroke (41) and possibly reduce the risk of infections that deteriorates the outcome of stroke patients. In line with this assumption, it could be shown that IVIg can even enhance microbial-specific immune responses in preterm infants (179) and do not increase mortality in sepsis (180). One clinical trial exploring the effect of IVIg in human stroke was already initiated (clinicaltrials.gov NCT01628055), but had to be stopped due to difficulties in patient recruitment.

## Conclusion

Taken together, there is growing evidence that the rapid activation of the immune system in response to acute sterile tissue damage can be detrimental for the affected organ. Particularly, postischemic inflammation following stroke has been investigated extensively and multiple preclinical studies emphasize beneficial IVIg effects in models of acute brain injury, i.e., ischemic stroke, spinalcord, and traumatic brain injury. The already established use of IVIg in various neurological diseases is a major advantage. Furthermore, available data suggest that IVIg are specifically modulating harmful inflammatory processes, without relevant immunosuppressive side effects.

In general, IVIg exert protective effects in autoimmune disease *via* multiple mechanisms. Similarly, in acute brain injury, it is most likely that IVIg protection is mediated by the interaction with different targets concomitantly, which merge to a mutual effect. In this context, immunomodulatory pathways are among the most promising candidates. IVIg can target microglia as resident immune cells of the CNS as well as immune cells from the systemic immune compartment and endothelial cells. Furthermore, it is important to mention that IVIg can stabilize the BBB and even facilitate direct neuroprotection. Eventually, it currently remains concealed if IVIg effects are Fc or F(ab)^2^ fragment dependent and if IVIg can modulate cells indirectly, which are not expressing Fc receptors in this context. Although the currently existing data are promising, further research is needed to gain more insight into protective IVIg-dependent mechanisms and to explore the therapeutic potential of IVIg in acute brain injury.

## Author Contributions

VT, TM, TA, and MG contributed to the concept design, writing, and final approval of the manuscript; VT drew the figures.

## Conflict of Interest Statement

The authors declare that the research was conducted in the absence of any commercial or financial relationships that could be construed as a potential conflict of interest.
